# Women Suffered More Emotional and Life Distress than Men during the COVID-19 Pandemic: The Role of Pathogen Disgust Sensitivity

**DOI:** 10.3390/ijerph18168539

**Published:** 2021-08-12

**Authors:** Yi Ding, Jie Yang, Tingting Ji, Yongyu Guo

**Affiliations:** School of Psychology, Nanjing Normal University, Nanjing 201097, China; yiding2017@hotmail.com (Y.D.); yangjie_163yj@163.com (J.Y.); yyguo@njnu.edu.cn (Y.G.)

**Keywords:** gender, anxiety, life distress, pathogen disgust sensitivity, COVID-19

## Abstract

The outbreak of the COVID-19 has brought upon unprecedented challenges to nearly all people around the globe. Yet, people may differ in their risks of social, economic, and health well-being. In this research, we take a gender-difference approach to examine whether and why women suffered greater emotional and life distress than men at the early stage of the COVID-19 outbreak in China. Using a large nationwide Chinese sample, we found that compared to men, women reported higher levels of anxiety and fear, as well as greater life disturbance during the COVID-19 pandemic. Importantly, that women suffered more was partly explained by their higher level of pathogen disgust sensitivity. Our findings highlight the important consequences of gender differences in response to the threat of the COVID-19 pandemic and suggest that policymakers pay more attention to gender inequalities regarding COVID-19 responses.

## 1. Introduction

In December 2019, a novel coronavirus disease, COVID-19, was discovered in Wuhan, Hubei Province, China, which rapidly spread throughout China and subsequently across the world. COVID-19 has a high infection rate and mortality, which led to disastrous outcomes for public health, economic development, and social polarization around the world [1,2,3]. As of 9 August 2021, there were a total of more than 200 million confirmed COVID-19 cases and 4.2 million deaths in approximately 220 countries and territories [4]. Clearly, nearly all people around the world experienced health, financial, and mental risks and stresses in the time of the COVID-19 pandemic, but they may not share the risks and stresses equally. For example, people in developing societies and those with lower socioeconomic backgrounds suffered more risks in terms of health, economic, and social well-being [5,6].

In this research, we aim to examine gender differences in reduced well-being during the COVID-19 pandemic. Although initial evidence suggests that women are at decreased risk of fatality and death due to COVID-19 than men [7,8], we predict that they might experience greater psychological distress during the COVID-19 pandemic. In particular, this research mainly focuses on whether and why women suffered greater feelings of anxiety, fear, and life disturbance than men at the early stage of the COVID-19 outbreak in China.

Previous research on infectious-disease outbreaks suggests that women suffered more social and psychological disadvantages from the outbreaks than men [9]. For example, during the outbreaks of Ebola and H1N1 influenza, women (vs. men) reported higher levels of anxiety, depression, job loss, and income decline [10,11,12]. They also suffered greater stigmatization, neglect, and domestic abuse in the times of these outbreaks [13]. Importantly, some initial evidence shows that women are more psychologically affected by the COVID-19 pandemic than men. For example, women in Italy, Spain, and Turkey reported greater anxiety, depression, and acute stress than men [14,15,16,17]. In China, women (vs. men) in the hardest-hit areas (i.e., Hubei Province) during the COVID-19 outbreak reported higher posttraumatic stress symptoms [18]. Given the above evidence, we predict that women rather than men would suffer greater emotional and life distress (Hypothesis 1).

**Hypothesis** **1** **H1.**  *Women rather than men would suffer greater emotional and life distress.*

Why do women suffer more than men from the pandemics? Very little research, however, has examined the mechanisms of such gender differences. Recently, some scholars argue that the COVID-19 social isolation and lockdown measures might cause women to be more vulnerable than men, as women take on more responsibilities of homeschooling and family caregiving as well as their increased risk of domestic violence, abuse, and neglect [19]. Here, however, we take an evolutionary pathogen avoidance approach to examine whether gender differences in sensitivity to pathogen-related disgust could explain greater emotional and life distress among women during the COVID-19 pandemic.

According to the behavioral immune system (BIS) theory, disgust is an evolved emotion that functions to facilitate behavioral defenses against cues of pathogens (e.g., bodily wastes), which possess the risk of being contaminated [20]. Pathogen disgust sensitivity is a term that is used to describe individual differences in the degree to which people feel disgusted toward cues of pathogens. Research found that individuals with higher disgust sensitivity experienced greater stress and anxiety disorders [21], especially during disease outbreaks. For example, during the 2009–2010 H1N1 “swine flu” and the 2014–2015 Ebola outbreaks, people with higher pathogen disgust sensitivity reported greater fear and anxiety in response to these disease outbreaks [22,23,24]. Notably, a recent study also found a positive correlation between pathogen disgust sensitivity and anxiety during the COVID-19 pandemic [25].

Importantly, a large body of evidence suggests that there is a replicable gender difference in pathogen disgust sensitivity [26,27,28]. Using a variety of measures, researchers found that compared to men, women scored higher in the Pathogen Disgust of the Three-Domain Disgust Scale [27,28] and reported more disgust with pathogen-related images [20]. From an evolutionary adaptive approach, women may have evolved gender-specific pathogen avoidance mechanisms both to protect themselves from sexually transmitted infections and the offspring, given that they suffer greater disease burden from these infections and have greater obligate parental investment than men [29]. Moreover, Al-Shawaf and his colleagues [30] provided six distinct possible explanations for why women have greater pathogen disgust than men: (a) a greater dependence of genetic vehicles on maternal investment; (b) a greater likelihood of transmitting infections to their offspring; (c) a greater role in protecting children from pathogens; (d) a greater role in food cleaning and food preparation; (e) stronger selective pressures for lower levels of disgust among men to facilitate mating; and (f) higher thresholds for blood, injury, and death among men because of selective pressures related to hunting and warfare. Building on this work and combining with the evidence that pathogen disgust sensitivity is positively associated with emotional and life distress, we thus predict that pathogen disgust sensitivity might mediate gender differences in emotional and life distress during the COVID-19 pandemic (Hypothesis 2).

**Hypothesis** **2** **H2.**  *Pathogen disgust sensitivity might mediate gender differences in emotional and life distress during the COVID-19 pandemic.*

The current research serves to test the two hypotheses using a large, nationwide Chinese sample at the early stage of the COVID-19 outbreak from January 2020 to February 2020, when the outbreak was salient and the China national government launched the nationwide lockdown policies. The goals of this research were to test whether women suffered greater feelings of anxiety, fear, and life disturbance than men during the COVID-19 outbreak, and whether pathogen disgust sensitivity could mediate gender differences in these emotional and life distresses.

## 2. Materials and Methods

### 2.1. Participants

The data were collected as part of a large research project aiming to investigate people’s social attitudes during the COVID-19 pandemic. A total of 1562 participants (719 females (46%), aged between 19 and 59, with a mean age of 31.31 years and SD of 8.14) were recruited online from Tencent (https://wj.qq.com; accessed on 31 January 2020), an online participant recruitment platform in China.

### 2.2. Measures

#### 2.2.1. State Anxiety and Fear

Participants completed the 6-item short-form of the state anxiety inventory (α = 0.87; e.g., “I feel upset”) [31] on a 7-point scale (1 = strongly disagree, 7 = strongly agree). We calculated the average score to represent the participants’ level of state anxiety during the pandemic, with higher scores indicating higher levels of state anxiety. Participants also rated their feelings of fear during the pandemic with one item (i.e., “I feel fear”) on a 7-point scale (1 = strongly disagree, 7 = strongly agree).

#### 2.2.2. Life Disturbance

Participants completed a 3-item questionnaire (α = 0.60; e.g., “I find it difficult to keep my mind on the work.”) [32] with ratings from 1 (strongly disagree) to 7 (strongly agree) to measure their life disturbance during the pandemic. We calculated the average score to indicate to what extent participants’ daily life has been disturbed by the COVID-19 pandemic, with higher scores indicating higher life disturbance levels.

#### 2.2.3. Pathogen Disgust Sensitivity

Participants completed a 7-item pathogen factor of the Three Domain Disgust Scale (α = 0.81) [27], which measures individual differences in pathogen disgust sensitivity. Participants reported how disgusting they find each of six items (e.g., “stepping on dog poop”) on a 0 (not at all disgusting) to 6 (extremely disgusting) scale. We calculated the average score to represent participants’ pathogen disgust sensitivity, with a higher average score indicating a higher level of pathogen disgust sensitivity.

#### 2.2.4. Demographics

Participants reported their age, gender, education level, and average monthly income. We assessed education level with six categories (1 = primary school or less; 2 = middle school graduate; 3 = high school graduate or equivalent education completed; 4 = junior college graduate; 5 = college graduate; 6 = postgraduate degree) and average monthly income with nine categories (1 = less than ¥1000; 2 = ¥1000 to ¥2000; 3 = ¥2000 to ¥3000; 4 = ¥3000 to ¥5000; 5 = ¥5000 to ¥8000; 6 = ¥8000 to ¥12,000; 7 = ¥12,000 to ¥15,000; 8 = ¥15,000 to ¥20,000; 9 = ¥20,000 or more).

## 3. Results

### 3.1. The Demographics Characteristics of Participants

Table 1 presents the demographic characteristics of our study participants. Women reported a lower average monthly income than men (*p* < 0.001), but no gender differences in age or education level (*p* > 0.05).

### 3.2. Common Method Variance Test

We conducted the Harman’s single-factor test to examine the common method variance [33]. The test revealed that the first factor accounted for 16.87% of the total variance and did not explain most of the variance (<40%). Thus, there was no obvious common methodological bias in this study.

### 3.3. Preliminary Analyses

Table 2 shows the correlations and descriptive statistics of all key continuous variables. As expected, pathogen disgust sensitivity positively correlated with state anxiety (*p* < 0.001), fear (*p* < 0.001), and life disturbance (*p* < 0.001) during the pandemic.

### 3.4. Gender Differences

Table 3 shows the gender differences in state anxiety, fear, life disturbance, and pathogen disgust sensitivity. As expected, compared to men, women reported greater state anxiety (*p* < 0.001), fear (*p* < 0.001), and life disturbance (*p* = 0.001) during the pandemic. Women also have higher levels of pathogen disgust sensitivity than men (*p* = 0.009). As noted earlier, women reported less average monthly income than men (*p* < 0.001). To examine the potential confounding effect of average monthly income, we conducted four linear regressions with gender as the predictor, and state anxiety, fear, life disturbance, and pathogen disgust sensitivity as the outcome variables, respectively, while average monthly income as the covariate. The results revealed the same gender effects on state anxiety, fear, life disturbance, and pathogen disgust sensitivity (*ps* < 0.004).

### 3.5. Mediation Analysis

**Figure 1:**
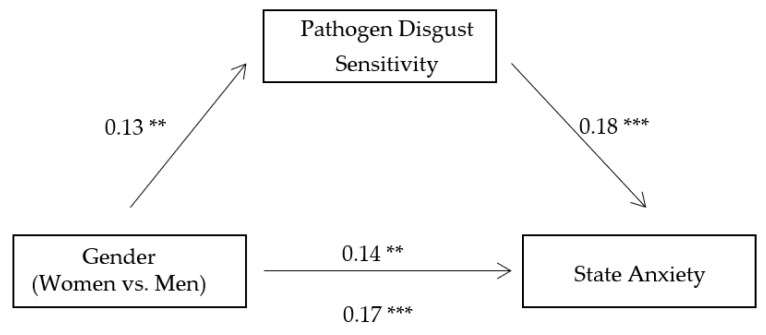
The mediating role of pathogen disgust sensitivity between gender and state anxiety. Note: Significance of the standardized regression coefficients is indicated (** *p* < 0.01 and *** *p* < 0.001).

**Figure 2:**
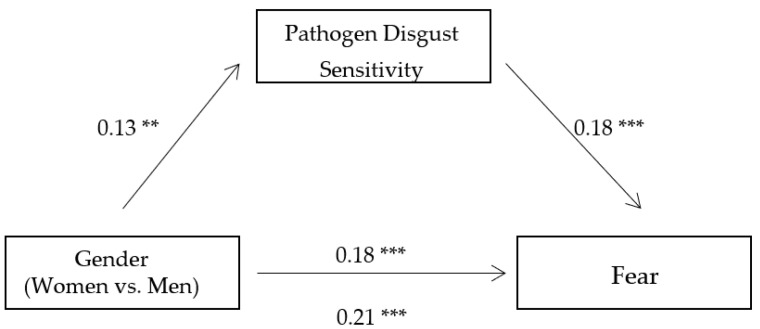
The mediating role of pathogen disgust sensitivity between gender and fear. Note: Significance of the standardized regression coefficients is indicated (** *p* < 0.01 and *** *p* < 0.001).

**Figure 3:**
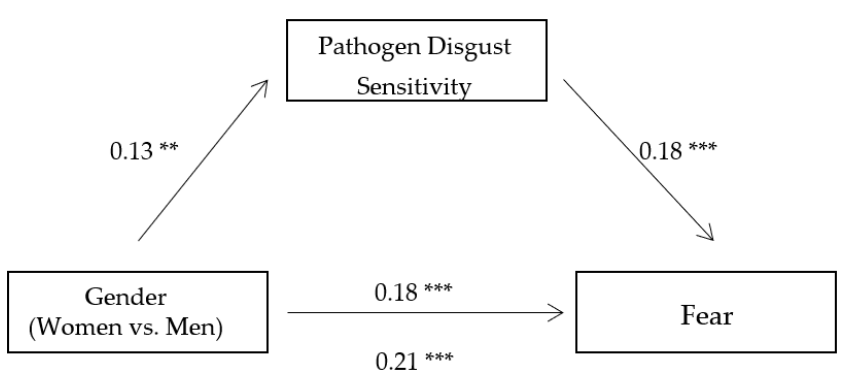
The mediating role of pathogen disgust sensitivity between gender and life disturbance. Note: Significance of standardized regression coefficients is indicated (** *p* < 0.01 and *** *p* < 0.001).

To further investigate the mediating role of pathogen disgust sensitivity between gender and state anxiety, fear, and life disturbance, we conducted three mediation analyses with state anxiety, fear, and life disturbance as dependent variables, respectively, using bootstrapping method based on 5000 bootstrap samples [34]. The results showed that pathogen disgust sensitivity significantly mediated the gender difference in state anxiety (B = 0.02, 95% CI = 0.01, 0.04, see Figure 1), fear (B = 0.02, 95% CI = 0.01, 0.04, see Figure 2), and life disturbance (B = 0.03, 95% CI = 0.01, 0.05, see Figure 3), respectively. These indirect effects were still significant when we included the participants’ age, education level, and average monthly income as covariates in the mediation analyses. These findings suggest that women experienced greater state anxiety, fear, and life disturbance than men during the pandemic partly because they have higher levels of pathogen disgust sensitivity.

## 4. Discussion

The outbreak of COVID-19 has brought upon unprecedented challenges to people’s health and economic livelihoods around the globe [35]. For example, evidence from Chinese and European samples found that the COVID-19 pandemic is like a storm of poor mental health and well-being, particularly for vulnerable groups [36,37]. In this research, we examined whether women suffered greater reduced well-being during the COVID-19 pandemic. Using a large sample at the early stage of the COVID-19 outbreak in China, we found that compared to men, women reported greater emotional and life distress, such as higher levels of anxiety and fear, as well as greater life disturbance. More importantly, gender difference in pathogen disgust sensitivity could partly explain this effect: higher levels of pathogen disgust sensitivity in women increased their emotional and life distress during the COVID-19 outbreak. Our findings highlight the important consequences of gender differences in response to the threat of the COVID-19 pandemic.

Some initial reports indicate that men are more physically vulnerable to the COVID-19 pandemic than women, showing significant gender disparities in morbidity and mortality [7,8]. Our research, however, found that women rather than men are more psychologically vulnerable to the COVID-19 pandemic. This finding is consistent with evidence on infectious-disease outbreaks, indicating psychological risks were more prominent in women than men during the pandemics [19]. In fact, previous research suggests that gender is a social determinant of health and well-being, especially women on average report to have worse mental health outcomes than men in daily life [38]. Our findings might suggest that the outbreak of COVID-19 may be deepening gender inequalities in terms of social, economic, and health well-being, considering COVID-19 has become a major stressor that has potentially severe negative consequences on our lives, in particular on the lives of women.

Importantly, our present research offers a novel perspective to explain gender differences in emotional and life distress during the COVID-19 outbreak. We found that compared to men, women have higher levels of pathogen disgust sensitivity, which makes them more sensitive to the threat of the COVID-19 outbreak, showing greater anxiety, fear, and life disturbance. This result is particularly important because it highlights that COVID-19 disproportionately affects women partly because of psychological motivations; women have greater disease-avoidance motivations, which contributes to their psychological distress response to the COVID-19 pandemic. We should note, however, evidence suggests that disease-avoidance motivations (e.g., disgust sensitivity) were also associated with fewer infectious illnesses [39] and greater health-protective behavior, such as social distancing and mask-wearing [40]. Thus, this might indicate the double-edged sword of pathogen disgust sensitivity in human health and help to explain why women are at decreased risk of fatality and death with the COVID-19 disease than men at the psychological level. Future work would benefit from examining the double-edged sword of pathogen disgust sensitivity directly and simultaneously.

Notably, some recent evidence suggests that pathogen disgust sensitivity could be changed depending on pathogen stress in the environment. For example, a recent study examined the Polish women’s pathogen disgust sensitivity before and during the COVID-19 outbreak and found that the COVID-19 outbreak increased women’s disgust sensitivity [41]. A recent behavioral genetic study also suggests that for women, disgust sensitivity was influenced by a combination of genetic (40.1%) and environmental (59.9%) factors [42]. Our present findings might raise the possibility that the COVID-19 outbreak increases pathogen stress in many societies, which causes greater disgust sensitivity for women, and therefore shapes their psychological distress response to the COVID-19 pandemic.

We should note that there may be some limitations in our present research. First, we did not measure participants’ professions or general health, which might be potential confounding variables that are associated with gender and psychological distress [43]. Second, as noted earlier, gender-based division of family responsibilities might be an important alternative explanation for gender differences in emotional and life distress during the COVID-19 pandemic. Future work could examine how it helps to explain our findings. Third, we investigated emotional distress during the COVID-19 pandemic only using general states of anxiety and fear. Future research would benefit from focusing on specific types of emotional distress caused by the pandemic context, such as COVID-19 health anxiety [44].

## 5. Conclusions

As COVID-19 continues to spread and impact our lives around the world, importantly, it has negatively affected people’s mental health and also created barriers for people to access mental health services due to the lockdown and social distancing measures; thus, policy responses to the COVID-19 pandemic require paying more attention to mental health needs [45,46,47]. Our present findings are relevant to this issue and reveal gender-based disparities in psychological distress during the COVID-19 pandemic. Women are more pathogen disgust sensitive, which causes them to be psychologically more strongly affected by the COVID-19 outbreak than men. Taken together, our findings suggest that mental health interventions and support need to have a gender perspective and pay more attention to the welfare of women during the COVID-19 pandemic, such as providing specific materials and messages related to women’s health care and well-being.

## Figures and Tables

**Table 1:** Demographic characteristics of our study participants (*n* = 1562).

Variable	Categories	Frequency	Percentage (%)	Cumulative Percentage (%)
Age	19–24	299	19.1%	19.1%
	25–30	592	37.9%	57%
	31–40	443	28.4%	85.4%
	41–50	175	11.2%	96.6%
	51–60	53	3.4%	100%
Gender	Women	719	46%	46%
	Men	843	54%	100%
Educational Level	Primary school or less	12	0.8%	0.8%
	Middle school graduate	47	3%	3.8%
	High school graduate or equivalent education completed	129	8.3%	12%
	Junior college graduate	453	29%	41%
	College graduate	757	48.5%	89.5%
	Postgraduate degree	164	10.5%	100%
Average Monthly Income	<¥1000	102	6.5%	6.5%
	¥1000–¥2000	103	6.6%	13.1%
	¥2000–¥3000	162	10.4%	23.5%
	¥3000–¥5000	391	25%	48.5%
	¥5000–¥8000	361	23.1%	71.6%
	¥8000–¥12,000	251	16.1%	87.7%
	¥12,000–¥15,000	82	5.2%	93%
	¥15,000–¥20,000	42	3%	96%
	>¥20,000	63	4%	100%

**Table 2:** Zero-order correlations among the key continuous variables.

Variable	M	SD	1	2	3	4
1. State anxiety	5.00	1.28				
2. Fear	4.72	1.69	0.72 ***			
3. Life disturbance	4.29	1.40	0.51 ***	0.51 ***		
4. Pathogen disgust sensitivity	4.45	0.99	0.18 ***	0.18 ***	0.23 ***	

Note: *n* = 1562; ***, *p* < 0.001.

**Table 3:** Gender differences in state anxiety, fear, life disturbance, and pathogen disgust sensitivity.

Variable	Women (*n* = 719)	Men (*n* = 843)	*t*	*p*	*d*
	M (SD)	M (SD)			
State Anxiety	5.12(1.23)	4.91(1.31)	3.32 ***	<0.001	0.17
Fear	4.91(1.59)	4.56(1.76)	4.06 ***	<0.001	0.21
Life disturbance	4.41(1.36)	4.19(1.43)	3.21 **	0.001	0.16
Pathogen disgust sensitivity	4.52(0.97)	4.39(1.01)	2.61 **	0.009	0.13

Note: *n* = 1562; **, *p* < 0.01; ***, *p* < 0.001.

## Data Availability

Data will be provided if requested to the authors.
